# Keeping Cavities Dry

**Published:** 1871-11

**Authors:** K. B Davis


### ARTICLE VII.

### *Keeping Cavities Dry.*

BY DR. K. B DAVIS.

Read before the Illinois State Dental Society.

To the operative dentist, who aspires to perfection in the insertion of his filling, the subject of controlling the saliva and keeping cavities dry, is one of pre-eminent importance.

No matter what form of gold we may use, whether foil or sponge, light or heavy, or whether we use hand or mallet pressure, absolute freedom from moisture is requisite to perfection in the fillings.

There are three sources from which the fluids of the mouth come, that annoy us in filling teeth, viz: from the salivary glands, mucous glands, and from the blood vessels of the gum when wounded in the course of the operation. In protecting cavities from the flow of saliva in operating upon the teeth, we must take into consideration the localities from which it comes. The saliva is principally secreted by three pair of glands: the parotid, the sub-lingual, and the sub-maxillary; and very nearly the whole of the secreted fluid passes into the mouth through two pair of ducts—the duct of Steno and the duct of Wharton. The duct of Steno passes from the parotid gland along the inside of the cheek, covered by the mucous membrane, and opens opposite the first superior molar. The duct of Wharton conveys the saliva secreted by the sub-maxillary gland, and the greater part of that formed by the sub-lingual gland, and opens on the side of the frænum of the tongue. To any one not familiar with these facts, the points at which the ducts open may be ascertained by examining the mouth at the points indicated.

Of the various means resorted to by the profession for keeping cavities dry, none have been so universally adopted as the napkin. This, in connection with spunk, may be used to protect simple crown cavities of superior molars from saliva very effectually. We can, ordinarily, secure dryness of these cavities by adopting the following method. Wipe the mucous membrane dry over the mouth of the duct of Steno, then lay directly upon this a pretty thick piece of spunk, about an inch square; then take a napkin and fold it cornerwise, and place the end of it directly upon the spunk, between the cheek and the gum, allowing it to pass back of the tooth to be filled, along the palatal surfaces of the teeth, to the anterior part of the mouth, letting a fold of it come down upon the tongue to protect the filling from the breath, and to catch any fragments of gold that may fall from the instrument. The spunk and napkin are to be held in this position by the fingers of the left hand, and if we will keep the mouth of the duct closed, a complete exclusion of moisture is secured.

In some cases it will require some pressure upon the napkin, while in others, very little, or none, will be required, the spunk adhering very firmly to the mucous membrane, and very effectually preventing any flow of saliva.

But can we protect cavities in the inferior teeth by the use of the above means? We certainly cannot, except in those favorable cases, that may be denominated "dry mouth." We can, with the addition of the tongue-holder to the spunk and napkins, fill small crown cavities in this class of mouths.

I direct the patient to place the point of the tongue against the roof of the mouth, as far back as possible. I then, with a piece of spunk, dry the mucous membrane around the mouth of the duct of Wharton, and immediately apply a piece of spunk, into which I have previously cut a slit to admit the frænum of the tongue, and packing it under and close to the sides of the tongue and gums. When this is done let the tongue come back into its place. Upon the tongue lay a smoothly folded napkin, and upon this adjust the tongue-holder. Now, on the side on which the tooth is to be filled, place a piece of spunk against the opening of the duct of Steno, on this a napkin, allowing a portion of it to come down upon the tongue to protect the filling from the breath, as in filling superior molars.

I presume that no one who pretends to operate upon the teeth is not already quite familiar with the use of the napkin and spunk, in protecting cavities from moisture, but I fear that there are many who have never learned to appreciate the value of a tongue-holder, a compressor for the duct of Steno, nor the *rubber dam*; although they have been in use by many in the profession for several years.

From the observation that I have made, it is my honest conviction, that not more than one dentist out of every ten in the country,—and perhaps some in our large cities—has ever attempted anything like the habitual use of the rubber dam.

New theories and new inventions, however meritorious, do not readily meet with public favor. It seems to be a difficult matter to give up old methods and theories of operating, even after far superior ones are fully demonstrated to exist. It is this fact which keeps many men all their lives on the track upon which they happen to start. When a new and better method is presented, perhaps they may slightly investigate it, try it a little, and because they cannot at once succeed, before they get familiar with it, better than by the old method, they at once abandon it, and go groping and stumbling along in the old way. Whenever a new method of practice is introduced, if the principle be correct, and it promises greater facilities in operating, with better results, it is the duty of every operator to examine it until its merits are fully appreciated.

To any one not familiar with the use of the rubber dam, the old method of protecting cavities from moisture may seem the better way; but when an acquaintance with the use of the results are thoroughly seen, which makes an ample return for all the time and labor spent upon it; and which will develope in a light never before so clearly seen, the imperfectness of the old plan of keeping cavities dry.

It is strange that men will constantly remain blind to the fact that it is a duty they owe alike to the profession, to themselves, and to the people, to at once familiarize themselves with the use of the rubber dam. We all know how difficult it is to keep a large majority of the cavities we are called upon to treat, dry, and the operator that is not in the habitual use of the rubber dam must exhibit a very limited influence in conservative dentistry; or else he must resort to the use of amalgam in a large majority of cases; which has a strong tendency to lower the operator's ability, and to depreciate the people's estimate of the profession at large.

The people *expect*, and as a *right*, *demand* of the operator, the use of every known means that is calculated to secure the very *best* results.

The rubber dam not only protects any and all cavities from the saliva, mucous secretions, and the breath, but it does much more than this. After we have it carefully adjusted on the tooth or teeth as the case may be, we feel ourselves complete masters of the situation, and we can then operate with perfect coolness and deliberation, without a single fear of an inundation, however difficult or long and tedious the operation may prove to be.

It is peculiarly serviceable in those large cavities requiring hours of time, taxing severely the patience of both operator and subject; it enables him to allow the patient a few moments rest occasionally, to spit out the extra flow of saliva; and all of this without fear of moisture coming in contact with the filling. Under these circumstances, what operator will not make much better fillings than he possibly can where he is laboring under constant fear that before he gets the filling completed it will be flooded, and that he will have to remove it all and commence anew, with the sad reflection, that a second effort may alike prove a failure.

I think I may say without a fear of successful contradiction, that at this day if any one still persists in refusing to familiarize himself with the use of the rubber dam, he very justly lays himself liable to severe censure, alike from the public and the entire dental profession.

We now come to a consideration of the application of rubber to the protection of cavities from moisture during the operation of filling.

The first requisite is to procure a good quality of rubber, and it should be pretty thick. Much of the rubber that was sold two or three years ago was entirely too thin, and consequently was worthless; but all that I have used for the last two years has proved to be of a very good quality.

The size of the piece to be used will vary, owing to the location where we wish to use it; generally a piece from three to eight or ten inches square will answer all indications.

Holes should be cut at the proper location in the rubber, in size about one-tenth the diameter of each tooth, and from one-eighth to a sixteenth of an inch apart. Carefully stretch the rubber until the holes are large enough to pass over the teeth, previously removing any deposits of tartar, should there be any present, so the rubber may draw tightly around the necks of the teeth and prevent any leakage. With a thin, blunt-pointed instrument work the edges of the rubber well under the free margins of the gums; there, from the natural shape of the tooth, it will in many instances remain; but in a majority of cases we will have a resort to a ligature to assist in its proper retention. We may use for this purpose saddlers' linen or silk, either of which should be well waxed before using. It is then placed around the tooth and with the blunt instrument above referred to, forced under the margin of the gum and worked well around the neck of the tooth, and then tied on the buccal side of the tooth in one knot, but with a double turn of the thread. If the teeth to which we wish to apply the dam approximate very closely, it is difficult to pass the rubber between them, but by carefully stretching it until the spaces between the holes become very small and thread-like we can generally succeed. However, if this does not suffice, we may insert a wedge between the teeth near the gum until a slight opening is made for the rubber to pass, when the wedge is to be removed. This process may sometimes become necessary on each side of the tooth.

In placing the rubber dam and ligature upon an upper or lower molar, Dr. Forbes' rubber dam applier may be used with much advantage and more pleasantly than we can use our fingers.

There are some lower molars that the dam cannot be retained upon by the ligature. In these cases we must resort to a clamp made especially for this purpose. Those I have in use were made by H. Spackler & Co., of St. Louis, and answer the purpose very well. In these cases where the cavities extend beyond the gum towards the neck of the tooth it is very essential to have the rubber worked beyond the margin of the cavity, or else we will fail in our main purpose and have moisture where we least desire it.

If the gum should be hypertrophied so as to interfere in forcing up the rubber, it may be removed with the lance or bistoury.

In the application of large pieces of the dam to the bicuspids or molars there is a strong tendency in the rubber to corrugate or wrinkle up into folds and thus obscure a good view of the cavity. To obviate this difficulty we may resort to the following method: The rubber being properly adjusted, cut four little holes in such localities as will be indicated in each individual case, and in each of these holes place a small wire hook, to which narrow pieces of tape or small cord are attached, letting these pieces of tape pass down under the chin or around the head, or both, as necessity may demand, and tieing them just firmly enough to hold the rubber down and entirely out of the way while introducing and finishing the filling.

The rubber dam has and will continue to work a mighty revolution in operative dentistry. A few years ago a large majority of operators from their inability to thoroughly keep cavities dry, were in the habitual use of amalgam and other cheap fillings, very many of whom have now learned the inestimable value of the rubber dam, and consequently are now enabled to preserve many of those priceless gems whose beauty and symmetry they were formerly in the habit of marring or ruthlessly destroying.

